# A Method to Assess Adherence in Inhaler Use through Analysis of Acoustic Recordings of Inhaler Events

**DOI:** 10.1371/journal.pone.0098701

**Published:** 2014-06-06

**Authors:** Shona D'Arcy, Elaine MacHale, Jansen Seheult, Martin S. Holmes, Cian Hughes, Imran Sulaiman, Deirdre Hyland, Conor O'Reilly, Senan Glynn, Thekra Al-Zaabi, John McCourt, Terence Taylor, Frank Keane, Isabelle Killane, Richard B. Reilly, Richard W. Costello

**Affiliations:** 1 Trinity Centre for Bioengineering, Trinity College Dublin, Dublin, Ireland; 2 The Department of Medicine, Royal College of Surgeons in Ireland, Dublin, Ireland; 3 Clinical Research Centre, Royal College of Surgeons in Ireland, Dublin, Ireland; 4 Dublin Centre for Clinical Research, Dublin, Ireland; 5 Vitalograph, Ennis, Co Clare, Ireland; University of Southampton School of Medicine, United Kingdom

## Abstract

**Rationale:**

Poor adherence to inhaler use can be due to poor temporal and/or technique adherence. Up until now there has been no way of reliably tracking both these factors in everyday inhaler use.

**Objectives:**

This paper introduces a device developed to create time stamped acoustic recordings of an individual's inhaler use, in which empirical evidence of temporal and technique adherence in inhaler use can be monitored over time. The correlation between clinical outcomes and adherence, as determined by this device, was compared for temporal adherence alone and combined temporal and technique adherence.

**Findings:**

The technology was validated by showing that the doses taken matched the number of audio recordings (r^2^ = 0.94, p<0.01). To demonstrate that audio analysis of inhaler use gives objective information, in vitro studies were performed. These showed that acoustic profiles of inhalations correlated with the peak inspiratory flow rate (r^2^ = 0.97, p<0.01), and that the acoustic energy of exhalations into the inhaler was related to the amount of drug removed. Despite training, 16% of participants exhaled into the mouthpiece after priming, in >20% of their inhaler events. Repeated training reduced this to 7% of participants (p = 0.03). When time of use was considered, there was no evidence of a relationship between adherence and changes in AQLQ (r^2^ = 0.2) or PEFR (r^2^ = 0.2). Combining time and technique the rate of adherence was related to changes in AQLQ (r^2^ = 0.53, p = 0.01) and PEFR (r^2^ = 0.29, p = 0.01).

**Conclusions:**

This study presents a novel method to objectively assess how errors in both time and technique of inhaler use impact on clinical outcomes.

**Trial Registration:**

EudraCT 2011-004149-42

## Introduction

Inhaled medicines have the advantage of direct application of drug to the lung with less systemic side effects. As with all medicines, failure to achieve a response to a clinically prescribed medicine may be the result of poor adherence. In the case of medication delivered via inhalers poor adherence may arise from non-use, haphazard or excessive use of medicine or poor inhaler technique. Temporal adherence is rooted in patient perceptions of the disease, belief in the medication, medication cost and access to healthcare [1], [2], while technique adherence is related to lack of or failure to remember instruction [3]. Several studies have highlighted that errors in inhaler technique may be as detrimental as the failure to use the inhaler [4], [5]. Regardless of whether it is from not using their inhaler or using it incorrectly, the consequences are poor clinical outcomes, wasted medications and higher healthcare costs [6]–[11].

Because there are such important consequences of poor adherence several approaches to assess inhaler adherence have been devised. At present, the most commonly used method in clinical trials is counting doses as read from the inhaler [12]. Other indirect methods include biomarkers such as drug levels and exhaled nitric oxide [13], [14]. However, these methods do not give a measure of everyday inhaler use. Electronic devices give a day-to-day measure of inhaler use but they do not assess inhaler technique [15]. Detection of technique errors is traditionally carried out through a face to face process with a clinician [16], [17]. However there is no way of assessing technique performance once the patient returns home. Hence, the limitations of all of these methods suggest that there is a need for a technology to longitudinally and objectively monitor both temporal and technique adherence.

The central aim of this work is to validate the hypotheses that technique errors can have an impact on clinical outcomes for users of inhaler medication. This was achieved by employing a device, which for the first time can track a user's adherence in both the technique and temporal domain over time. Measures of adherence over 12 weeks are determined by processing the audio files, which are created every time the subject uses their inhaler, for evidence of good or poor inhaler use. These values of adherence are then correlated with changes in clinical outcomes to assess the validity of assessing adherence in this manner.

## Methods

### Adherence Monitoring Device

**Figure 1:**
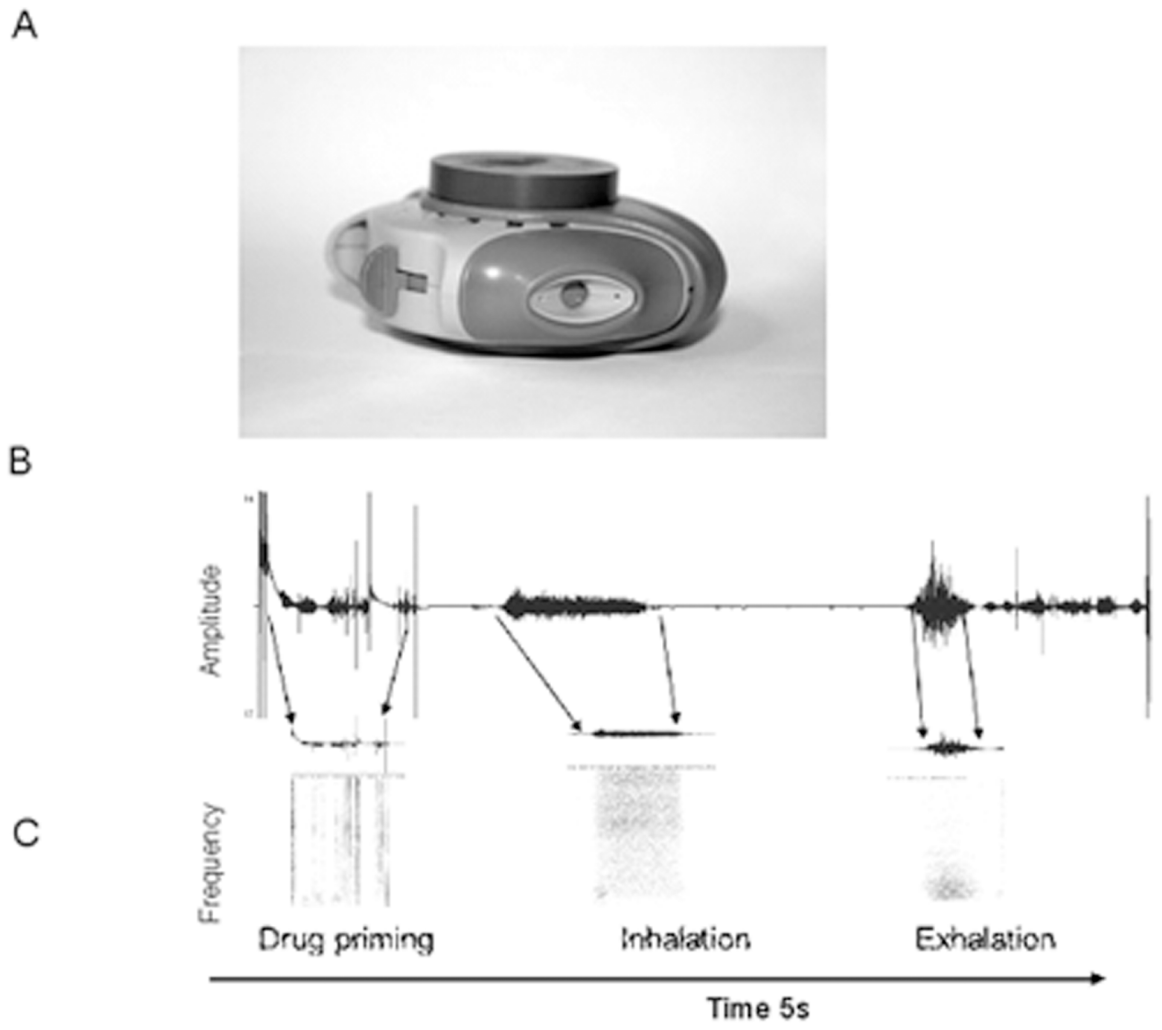
The audio recording device, attached to the Diskus inhaler is shown in (A). In (B) the amplitude of the audio associated with an inhaler being used is shown, in (C) the corresponding audio is shown in the frequency domain. From analysis of the audio the clear differences in the features of each of the steps is shown. After fully opening the device, which starts electronic recording, the first critical step is the lever movement to blister the drug. This step is characterized by a short burst of energy lasting approximately 20–30 ms with a high frequency content (∼2 kHz) preceded by a short burst of lower frequency noise (∼1 kHz). Prior studies have shown that there is a difference in spectral components in the frequency domain between inhalations and exhalations an exhalation has a sharp increase in amplitude that tapers off with time and the power of exhalation decreases exponentially from 2 kHz to 500 Hz while the spectral power for inhalations are higher and they have a low increase in amplitude compared to that of exhalations.^18^

The Inhaler Compliance Assessment (INCA) device consists of a microphone, a battery, solid-state memory storage and a microprocessor for recording audio. The prototype device was attached to a Diskus inhaler Figure 1A. Recording is initiated by opening the inhaler and finishes when the inhaler is closed. An electronic real-time clock marks the time the recording is made and this is stored as part of the file's metadata. The audio is recorded at a sampling rate of 8 kHz with an 8bit sampling resolution, Figure 1B. An initial 49 patients used INCA Version 1. This group of devices had a failure rate of 13%. Additionally, in the last two weeks of the study, in these patients, 6% of device batteries failed, however there was data available for the first two weeks. Modification to the battery and firmware in Version 2 reduced the subsequent device failure rate to 3 of 51 devices (6%).

Each participant was recruited for three consecutive months, at the beginning of Month1 they were given an INCA enabled Diskus inhaler and at the end of that month they returned to the clinic and received a replacement. During their return visit patients had their inhaler technique assessed and corrected if required. The audio files on the INCA device were downloaded and processed for subsequent analysis. The protocol for this trial and supporting CONSORT checklist are available as supporting information; see Protocol in File S1 and CONSORT S1.

### Classification of inhaler events

The package instructions accompanying a Diskus inhaler describe the steps required for its correct use. Table 1 and Figure 1B demonstrate how each of these phases can be identified visually from a display of an acoustic recording created by the INCA device. Visual and audio analysis was carried out using the audio processing software Audacity, (http://audacity.sourceforge.net).

**Table 1:** Comparing Inhaler steps to INCA device Function.

	Inhaler Checklist	INCA Device Functionality	Impact on Technique Adherence
1	Use thumb or finger in thumb grip to open device until the mouthpiece appears	INCA device starts recording	Critical
2	Keeps Diskus horizontal	Experiments reveal that only shaking will remove drug from inhaler	Non-Critical
3	Slide lever once until it clicks	Blister sound identified from audio signal (“drug priming” in figure 1B)	Critical
4/5	Breathe out fully When breathing out fully does so away from Diskus	Any exhalations in the direction of the inhaler and be observed in the audio recording. An exhalation after the drug has been blistered is a technique error (Figure 5A)	Critical
6	Presses lips tightly above & below mouthpiece opening	Inhalation identified from audio signal (“inhalation” in Figure 1B)	Critical
7	Breathes IN QUICKLY, filling lungs with medicine	Inhalation identified from audio signal (“inhalation” in Figure 1B)	Critical
8	Holds breath for at least 5 seconds (with or without Diskus in mouth)	This can be detected in the audio signal (“exhalation” in Figure 1B)	Non-Critical
9	Removes Diskus before breathing normally	Recording ends	Critical
10	Closes Diskus by placing thumb or finger in the thumb grip & sliding it closed	Recording ends	Critical

### Ethics Statement

This observational study was approved by the Beaumont Hospital Research Ethics Committee 09/58 and the Irish Medicines Board C10026#0 and registered with EudraCT (number 2011-004149-42). Written informed consent was obtained for all participants in the study. This study was not registered as a clinical trial as this was considered an observational study of the technology employed.

### In vitro testing to acoustically identify the steps involved in inhaler use

**Figure 2:**
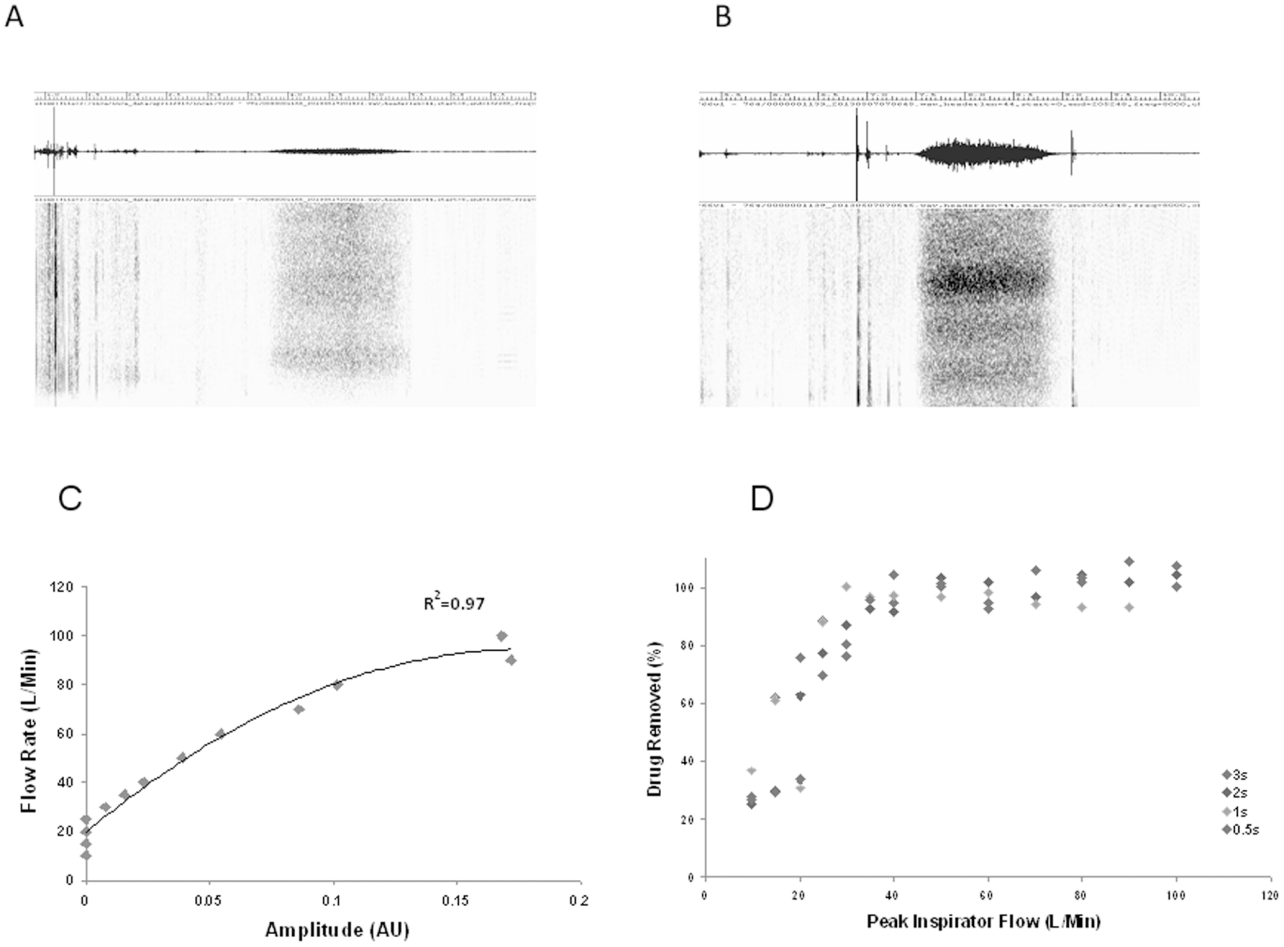
The amplitude and corresponding spectrogram of an individual with a weak inhalation is shown in (A). In (B) the amplitude and corresponding spectrogram of another individual with a strong inhalation is shown. In (C) the relationship of the amplitude of inhalation to peak inspiratory flow rate is shown, there is a strong relationship between these two variables, r^2^ = 0.97. In (D) the relationship of amplitude of inhalation to drug removal is shown.

Studies showed that there was variability in the amplitude of acoustics when individuals inhaled, Figure 2A and B. We quantified the relationship of the acoustics of inhalation with peak inspiratory flow rates and with drug delivery. The technical details of these experiments are presented in “Objective measures of acoustic profile of inhalation and exhalation” in File S1. The amplitude of inhalation was closely related to the inspiratory flow rate, R^2^ = 0.97, p<0.01 Figure 2C. The amplitude of inhalation was also related to the weight of drug extracted from the inhaler, Figure 2D. This allowed us to objectively assess if there was a critical error in inhalation, if the median amplitude was <0.016AU (corresponding to a flow rate of 30 L/min) then such an event was classed as an inhalation error.

As many files contained evidence of exhalation, after the lever had moved to prime the inhaler, we performed studies on the effect of exhalation on drug availability. In these it was shown that an effort of exhalation above 1 dB, dispersed more than 50% of the drug. Hence, if an exhalation occurred after the lever had moved then the event was considered to be an error in inhaler technique.

### Clinical testing

**Figure 3:**
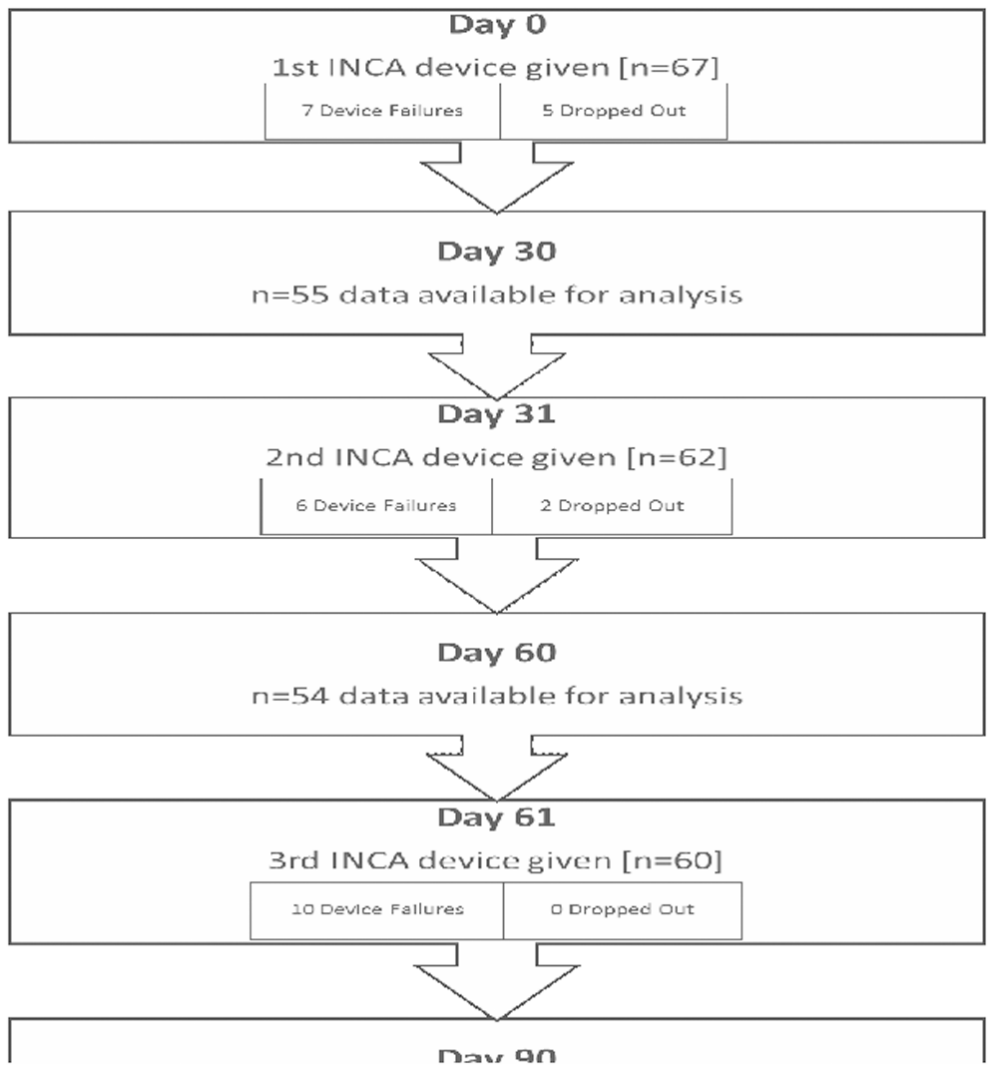
A consort diagram for the 69 patients who participated in the study is shown.

A salmeterol/fluticasone Diskus inhaler was chosen for clinical studies with the INCA device. The study inclusion and exclusion criteria are shown in Table 2, a consort diagram is shown in Figure 3.

**Table 2:** Inclusion and exclusion criteria for participant recruitment.

Inclusion	Exclusion
Capable of understanding and willing to provide voluntary informed consent before any protocol specific procedures are performed	Be females of childbearing potential who are pregnant, or intend to become pregnant, or are not using adequate contraceptive methods
Clinical diagnosis of asthma whose recent clinical condition indicates ongoing need for combination therapy.	Have used any investigational product or device within 3 months of the enrolment visit.
Age 18 years or older at time of consent.	Have known previous sensitivity to salmeterol/fluticasone.
Capable of understanding and complying with the requirements of the protocol, including ability to attend for all required visits.	Have a known significant (in the opinion of the investigator) concurrent medical disease.
Able and willing to take inhaled medication.	
In the opinion of the investigator suitable for use of a salmeterol/fluticasone inhaler or already using a salmeterol/fluticasone inhaler.	

Sixty-nine patients were given an INCA enabled Diskus inhaler. The baseline characteristics of the patients are shown in Table 3. The patient cohort consisted of 30 males (43%) and 39 (57%) females who were prescribed either 50/250 mcg or 50/500 mcg doses of salmeterol/flutocasone to be delivered via a Diskus inhaler. Demographic data such as age, height and weight as well as asthma related measurements such as Asthma Quality of Life Questionnaire (AQLQ) and Peak Expiratory Flow Rate (PEFR) are collected at the initial visit to the clinic. The clinical data relating to the subjects, from month 1 to month 3 of the study, are shown in Table 4.

**Table 3:** Baseline details of the original study cohort; BMI  =  Body Mass Index, AQLQ  =  Asthma Quality of Life Questionnaire, PEFR  =  Peak Expiratory Flow Rate.

	Mean	Range	Confidence Interval
Age (years)	46.78	(14.5–83)	42.74–50.81
Sex	Male Female	30 39	
BMI m/kg^2^	28.5	(15–55)	26.48–29.98
Number of exacerbations in past year	4.45	(0–12)	2.71–4.91
Number of episodes of steroid use in past year	2.92	(0–12)	2.16–3.68
AQLQ score	4.06	(1.1–6.7)	3.74–4.38
PEFR (L/min)	413	(155–800)	378–447
			

**Table 4:** Recorded average clinical measures over study period; AQLQ  =  Asthma Quality of Life Questionnaire, PEFR  =  Peak Expiratory Flow Rate.

Values	Baseline	End of study	*p value*
AQLQ	4.06 (1.13–6.73)	5.01 (1.20–7.00)	*0.0005*
PEFR (L/min)	417(155–800)	428(180–700)	*0.211*

All participants were recruited from Beaumont hospital and an initial target of 50 participants was set. Initial participant recruitment was from January 2011 until June 2012. Due to device failures a second round of recruitment was required to meet the target number of 50, this was carried out from April 2013 until June 2013. Any participant who experienced more than two device failures over the course of their study period was not included in the analysis. In June 2013 data from 51 participants who had at least 2 months of data from INCA devices was available for analysis.

As this was a validation study the primary endpoint was to demonstrate that the device recordings were equivalent to the dose counter, the current gold standard method. Exploratory secondary aims were to quantify the prevalence of errors in technique and compare the rates of adherence, when assessed in the time domain alone and when technique and time of use were assessed together. Other analysis included a comparison of adherence rates between those subjects who demonstrated clinical improvements in AQLQ and PEFR.

### Statistical methods

**Figure 4:**
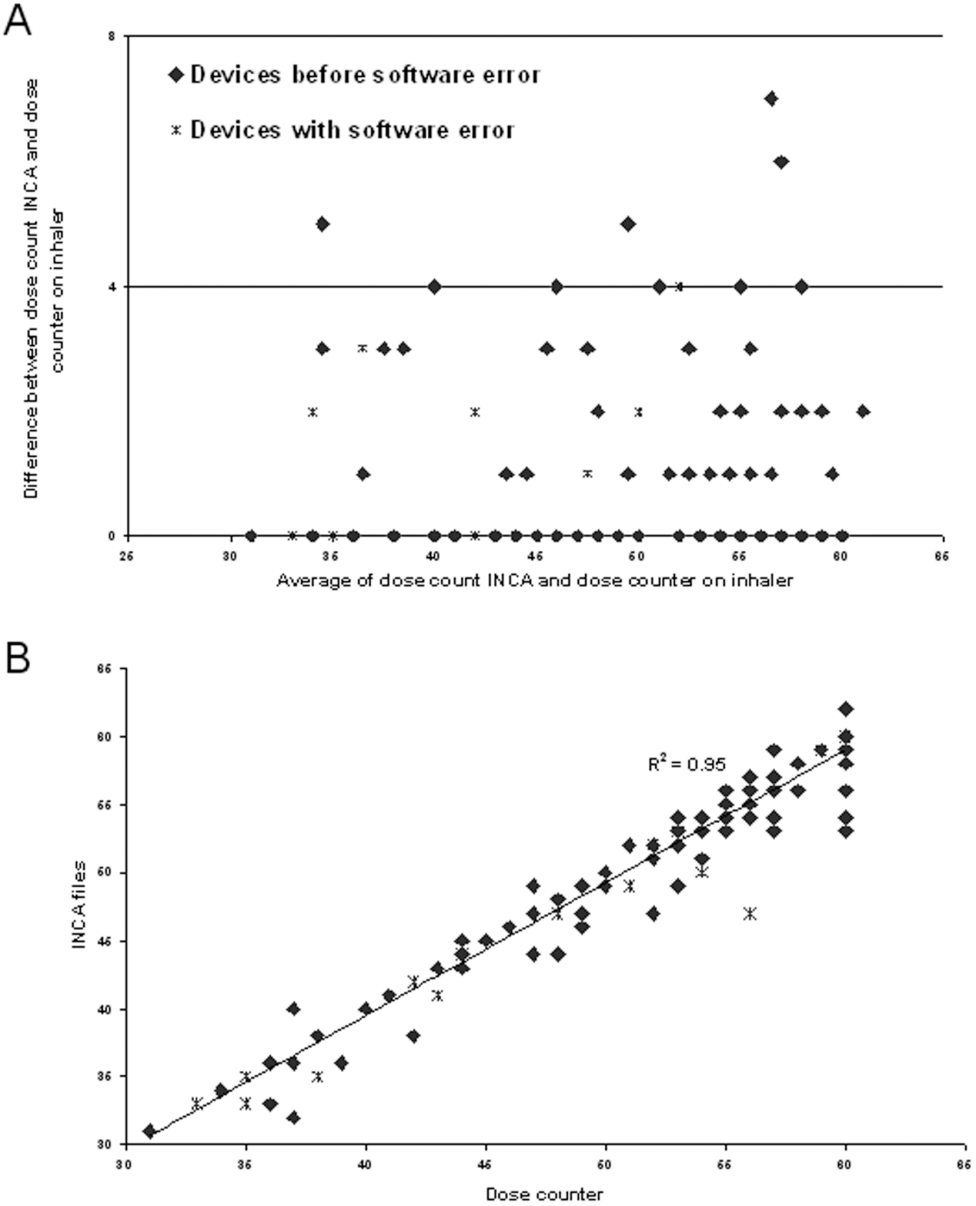
A Bland Altman plot showing the relationship of the doses taken, recorded by the dose counter on the Diskus and the number of audio files logged on the metadata of the INCA device is shown in (A). In (B) the same data is displayed as a correlation of the doses taken to the number of audio recordings.

**Figure 5:**
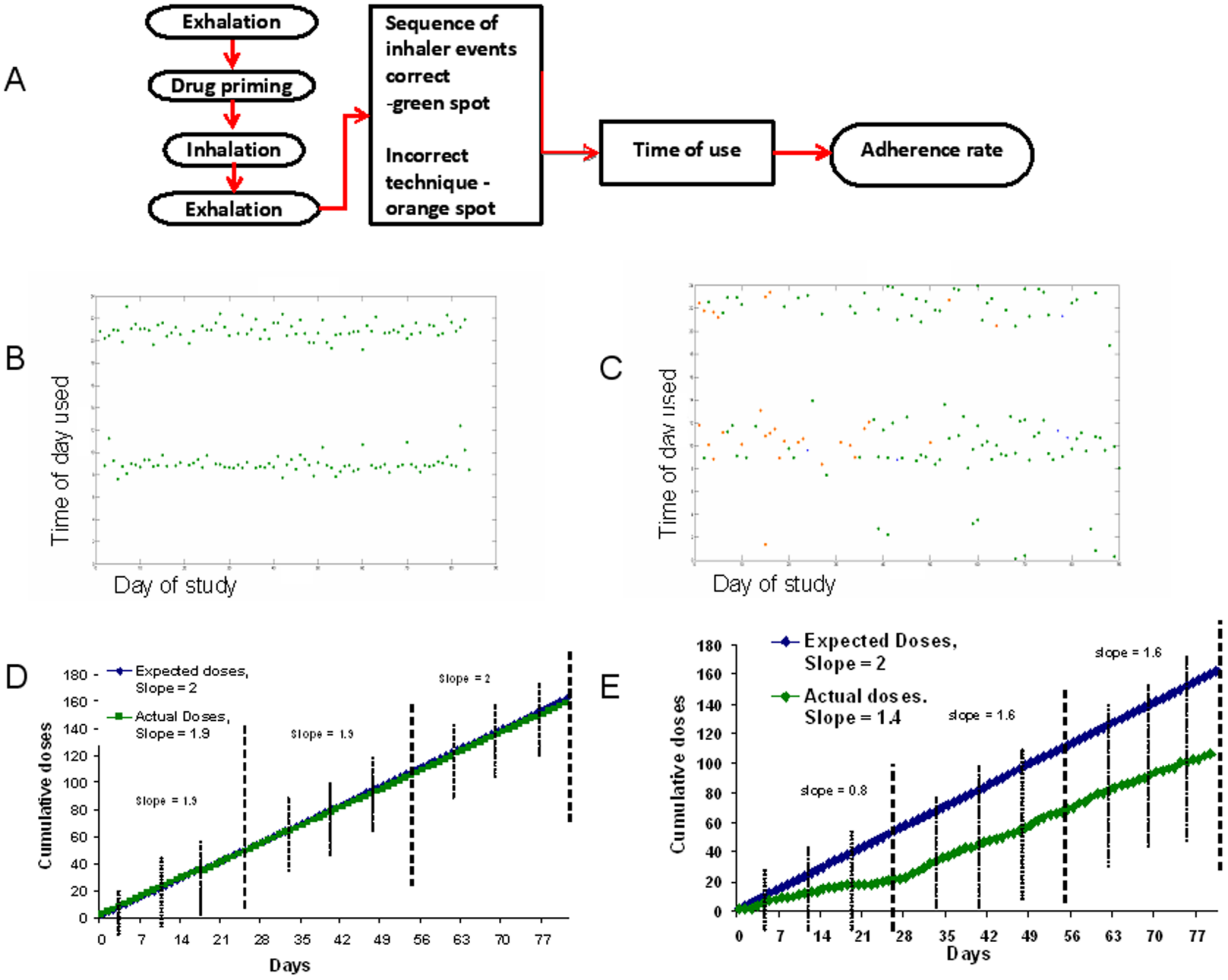
The decision algorithm, outlining the steps taken to analyse each recording, is shown in (A). In (B) and (C) the data from two individuals over 90 days are shown on the X-axis, and the time of use on the Y-axis. If the sequence of events required for correct inhaler use is performed, with sufficient energy of inhalation, then the event is displayed with a green spot. If there is a critical error, then the event is displayed with an orange spot. In (D) and (E) the weekly rate of adherence incorporating the time and technique are shown. It should be noted that while both users took their inhalers very regularly, the individual in (E) had poor technique in the first month, which impacted the true rate of inhaler use.

The number of INCA audio recordings, which contained acoustic evidence that the drug was dispensed, was compared to the number of doses taken by the user, quantified by the mechanical counter on the inhaler. The level of agreement between these two methods of dose counting was investigated using Pearson's correlation coefficient and a Bland Altman plot, figure 4A. A Bland Altman [18] plot is a difference plot used to demonstrate the agreement between two types of measurements. In order to determine the rate of adherence the cumulative number of correctly taken doses was plotted against the cumulative number of prescribed doses (2 per day) in 7 days. A regression line was fitted to the plot and the slope of this line was calculated. This slope is compared to that of the perfect adherence regression line, i.e. slope = 2, to assess deviation from perfect adherence. The slope of the line is calculated from 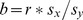, where r is the correlation between X and Y, s_x_ is the standard deviation of the X and s_y_ is the standard deviation of the Y, see figures 5D & E. Slopes of regression lines, plotting adherence rates over time, were compared using ANCOVA to determine if the slopes of the lines are statistically different.

## Results

### Device recordings and dose counter

In this study 69 patients used three separate INCA devices attached to their salmeterol/fluticasone inhaler. From the dose counters there were 8416 doses taken, giving an average adherence of 89%. From the audio files downloaded from devices that successfully recorded for the full month there were a total of 7391 inhalations. There was a close correlation between doses taken and audio files recorded on the INCA device (Figure 4), (R^2^ = 0.95 p<0.01). Manual classification was carried out by two separate individuals for all patients. This involved visually inspecting the audio spectrogram and listening to the audio files, to identify each procedural step in inhaler use [19]. Inter-observer reliability gives a kappa score of 0.7. Automation of the temporal and technique inhaler use was then addressed by developing a processing acoustic algorithm, which showed a sensitivity of 95%, specificity of 94% and an accuracy of 89% in detecting inhalations and drug priming compared to manual classification [20].

### Adherence in the technique domain

**Figure 6:**
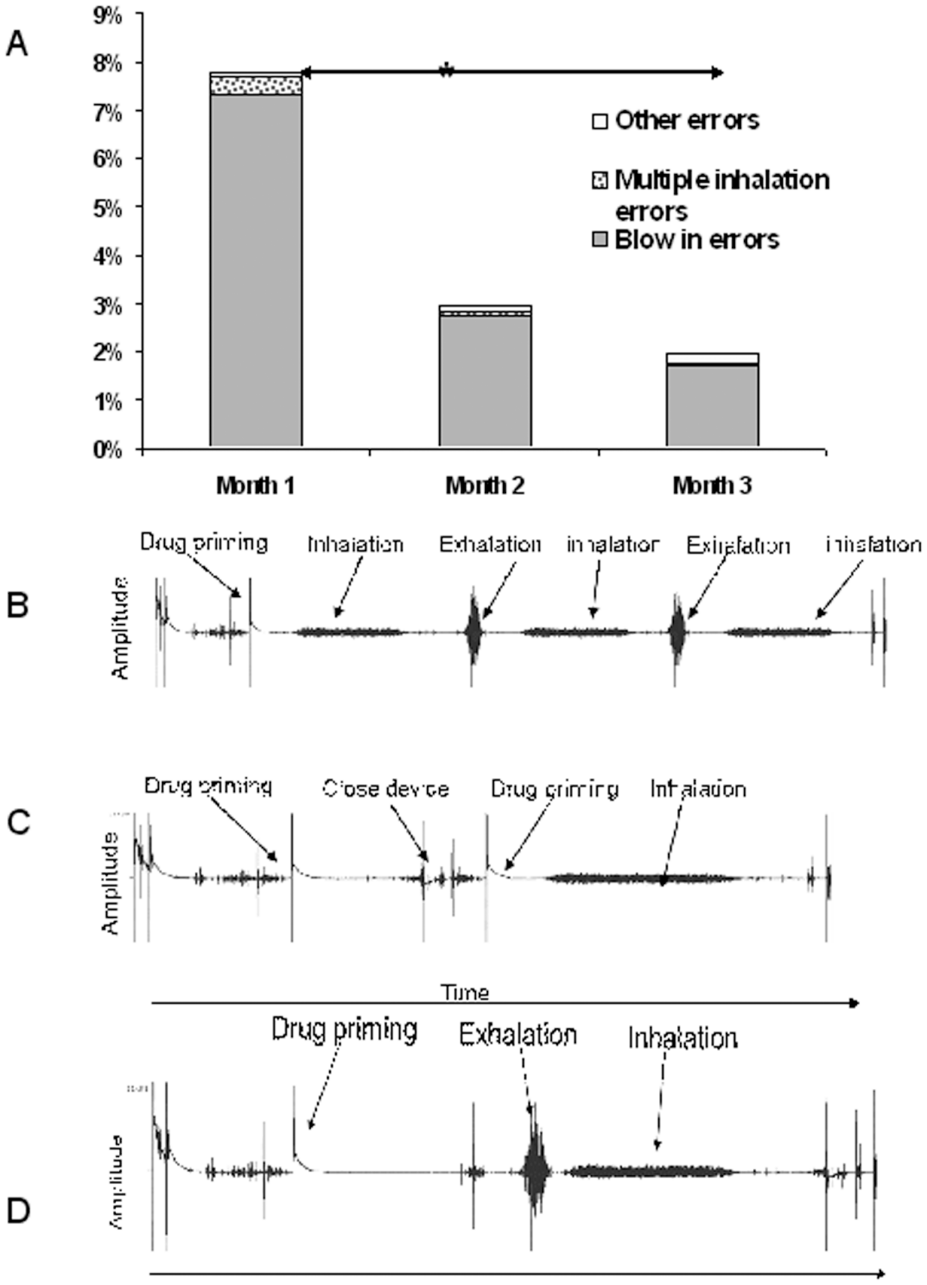
The percentage of all recordings with an error in use is shown in (A). Before participating in the study all patients had demonstrated that they were proficient in inhaler use, nonetheless at the end of month 1, 10% of all inhalations had a critical error. Over the next two months there was a significant reduction in the number of errors made by the patients, (*, p<0.05). In (B) a recording of an individual taking two doses, one after the other is shown. In (C) a recording of an individual moving the lever back twice, effectively wasting a dose. In (D) an example of a recording where the user exhales into the mouthpiece after priming and before inhalation

The frequency of all errors and individual errors are presented in Figure 6. In total 599 (8%) of total inhaler events (n = 7391) were classed as containing at least one technique error. Blowing into the device prior to an inhalation was the most common technique error observed in this study. A number of other errors were detected, such as breathing in and straight out Figure 6B or moving the lever twice, Figure 6C or taking 2 doses one after another Figure 6D. These were infrequent errors and so were classified as “inadequate technique”. Of the 8 (17%) subjects from month 1 who had more than 20% technique errors, 6 reduced their errors to less than 10% in subsequent months, after 3 consecutive episodes of training. On the other hand three patients did not reduce the proportion of errors observed in their recorded audio over the course of the three months. An additional three subjects increased their errors over the 3 months. The most significant example was from an individual who increased errors from 8% in month 1 to 55% in month 3. This was due to addressing step 4 in the inhaler checklist, i.e. “emptying lungs before inhaling” (see Table 1). In this case the subject, who was not completing this step before education, was now exhaling but into the inhaler.

### Adherence in the time domain

The electronic process of recording the audio file also creates a time log; this was used to calculate the temporal adherence. The analysis of the recordings of the overall temporal adherence for all doses taken by the study participants is shown in Tables S1 and S2 File S1. A graphical display of inhaler use by two different patients, generated from the metadata from the INCA device, is shown in Figure 5B and C.

### Combined adherence

Assuming that technique errors can result in a dose of medication not being optimally delivered to the lungs, we combined the temporal and technique data to assess this composite effect, see Table S1 and S2 in File S1. Figures 5 C and E in particular demonstrate the effect technique errors can have on overall adherence. A significant number of technique errors (yellow dots) can be seen in the data for week 1. Although the patient roughly took the recommended number of doses over this period, the technique errors significantly affected adherence rate reducing it from an expected rate of 2 to 0.8. It can then be seen that technique errors improve in subsequent weeks, and adherence rate increase to a more acceptable value, 1.6. In month 1, when considering technique errors the same as missed doses the rate of adherence was 10% lower than when just the temporal adherence was considered alone.

### Relationship of clinical progress to adherence

**Figure 7:**
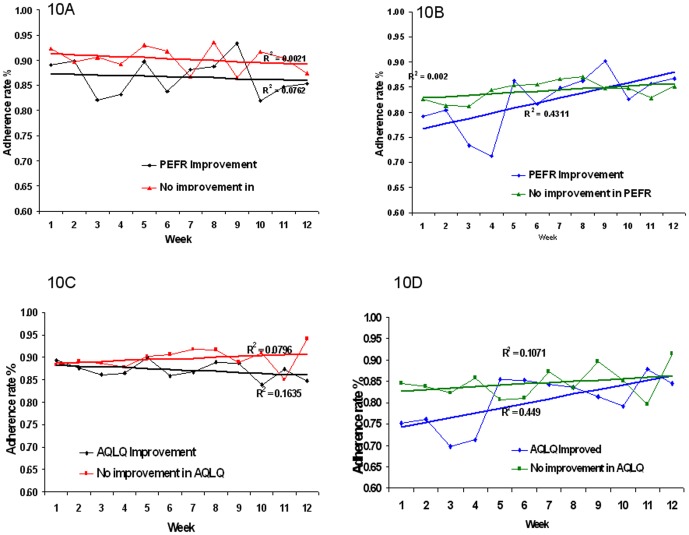
In (A) the rate of adherence by time of use for patients who improved by the minimum clinically important difference in AQLQ are shown in red and those who did not is shown, in black. Time of inhaler use did not relate to changes in clinical status. In (B) the adherence rate including the time and technique of use is shown. Those who had at least a minimum important clinically difference in AQLQ over the observation period are shown in blue, while those who did not are shown in green. There was a significant relationship between the rate of adherence and the outcomes in AQLQ when both time and technique were assessed. In (C) the rate of adherence by time of use for patients who changes in PEFR is shown, the red line is those who had a trend of improved PEFR and those who did not is shown, in blue. In (D) the adherence rate including the time and technique of use is shown, there was an association of the improvement in PEFR with increased inhaler use, blue and those who did not, green.

Among the study participants there was an overall significant increase in AQLQ (p<0.001) over the 12 weeks of study, see Table 4. An exploratory analysis was performed to relate the rate of adherence in those who achieved at least a minimum clinically important improvement in AQLQ of 0.5 (n = 28) and those who did not (n = 18) [21]. No statistical difference was observed between the two groups for age or baseline PEFR, however there was a statistical difference in the BMI's and AQLQ scores for the two groups (p = 0.04 and 0.02 respectively). For this analysis temporal adherence only considers whether two doses were taken in a day. When plotting rate of inhaler use (Figure 7A) no relationship between the rate of adherence and clinical outcomes was observed. When data on time and technique was combined a correlation between the AQLQ-improvers group and this combined adherence rate was observed; with a downward trend in adherence for the non-improving group, (p = 0.017) and an upward trend from improvers (p = 0.02), Figure 7B. The same analysis was carried out comparing those with improvements and no improvements in PEFR, n = 20 and n = 28 respectively. There was no statistical difference in the age, BMI, initial PEFR or AQLQ scores between these two groups. No relationship between adherence, calculated simply by time of use between improvers and decliners, was observed however using a composite rate of adherence showed a distinction in the trend between improvers and non-improvers in terms of their PEFR rate, p = 0.016, (Figure 7 C & 7D).

## Discussion

This study reports a novel method to assess the use of an inhaler by an individual. The technology involves an audio recording device and a methodology which provides objective evidence of inhaler use and a novel way to calculate adherence. The clinical significance of this approach is that when both the technique of inhaler use and the time of use are considered together this provides a more objective relationship of the patient's use of an inhaler to their outcomes.

We undertook this study because there is a need to have a technology that quantifies when and how an inhaler has been used, since clinicians often cannot distinguish if the progression of a condition such as asthma is influenced by adherence to therapy or deterioration in the condition. As a means of assessing inhaler use, audio recordings have several advantages. Electronic recordings are time stamped, so that the time of use can be assessed and the technology involved in audio recording devices has become smaller and more robust. Furthermore, audio can be analysed in the time and frequency domains, meaning that objective quantifiable features corresponding to each step of the use of the inhaler can be identified and extracted for analysis. We initially used human raters in the assessment of the steps of inhaler use and then devised a method to perform the process automatically. We compared these automated assessments with those made by trained human assessors. The automatic signal processing method has a high sensitivity and specificity [22]. This means that analysis can be performed rapidly so that the information can be presented to an individual in real time.

The traditional methods of assessing adherence with dose counters or electronic recordings reflect when the inhaler was used but they do not assess how it was used. In this study there was no relationship between temporal adherence and clinical outcomes. It was only when errors in inhaler use and time of use were combined that a relationship between changes in adherence and changes in outcomes was identified. This means that it is important to know both when and how an inhaler was used.

Analysis of the audio recordings revealed some important findings that are not easily identified by the traditional method of direct visual assessment. Direct visual assessment simply shows that the individual is competent in using their inhaler but cannot guarantee that the process is followed in the individual's home environment. In this study all patients were fully trained and judged to be competent in the use of their inhaler at the start of the observation period but nonetheless 17% of patients had more than 20% errors during the initial month. This indicates that errors do persist despite one-to-one education. It has been shown previously that repeated education leads to better inhaler technique [23] and this was also the case in this study. In fact it required three consecutive training sessions, over 3 months, to reduce errors in technique to <5% of all inhaler events. In this observational study eliminating these errors corresponded to the observed improvement in asthma quality of life. The majority of patients who made errors did not do so every time suggesting that these errors reflect carelessness rather than poor proficiency. Despite repeated training three patients did not improve their technique and three actually developed errors in their inhaler technique. Hence, while patients can be seen to be competent in using their inhaler many persist in making mistakes, which have direct clinical impact, when they use their inhaler outside of the training setting. Given that the acoustic algorithm can process the audio data in real-time we can incorporate this information into a tailored training program based on an individual's own technique errors. By displaying information on rate of use along with information on clinical symptoms and PEFR, as shown in Figure S1, we can provide an individual with a greater insight into the relationship of inhaler use to their own outcomes. A clinical trial is underway to assess if providing such feedback improves clinical outcomes more than generic inhaler training.

In vitro, studies have demonstrated a strong relationship between the acoustics of inhalation and the inspiratory flow rate. Devices such as the peak inspiratory flow rate meter can assess if the patient can achieve a certain flow but they cannot assess the day-to-day flow rate achieved by the patient. Most patients in the study easily achieved an adequate peak inspiratory flow. However other patients, for example those with COPD, may not achieve adequate flow rates, which will limit the effectiveness of their inhaler [24].

The most common error made by patients was an exhalation into the inhaler after the lever had been deployed to activate the drug. This error disperses the medication away from the mouthpiece and so reduces the quantity of drug available for subsequent inhalation. Another component of this error is the introduction of water vapour to the mouthpiece which can also impact drug delivery (further details on the impact are discussed in the accompanying second manuscript). In this study analysis of the audio files allowed us to objectively identify when this error occurred. For most patients this seemed to occur because they do not recognise the importance of the instruction “exhale fully away from the device”. Having objective evidence from the audio recordings ensures that these errors are identified. Development is currently ongoing to automate the classification of inhaler events. This will result in a very usable system for monitoring patients in a clinical environment. Technique errors are specific to this type of inhaler; however there are many types of inhalers available, each with their individual potential technique errors. For example the orientation of the diskus inhaler is often considered a potential source of technique error. It is recommended that the inhaler is kept horizontal during use. Preliminary experiments demonstrated an inhaler held at 45° or 90° will only loose between 0 and 5% of drug present in the inhaler. It is only with shaking or tapping the inhaler when help at 90° that drug will be dislodged from this inhaler. This study presents a successful framework for identifying technique errors and establishing objective methods of classification of these errors

There are several limitations to the study. Firstly, the rates of adherence were very high, compared with other studies of adherence in asthma [1], [6], [8], [11], [14], [15]. The study was performed in the setting of a formal research setting. In other preliminary studies, in general practice, on hospital wards and in their own home following exacerbations of COPD with this device, we have shown much lower levels of adherence [25],[26]. In the current study the participants were fully aware of the purpose of the study, which was to assess inhaler use over a period of time. Therefore, the usual limitations of this type of study design apply; including the Hawthorne effect, knowing that there adherence was being assessed may well have altered the rates of adherence. The results do indicate that despite high levels of temporal adherence many patients had errors in technique which when they were addressed were associated with improvements in their symptoms. Hence, repeated training in inhaler technique has direct clinical effects.

In summary, we describe the use of audio recordings to provide objective longitudinal evidence of inhaler use. This information can provide an insight into the relationship between inhaler use and an individual's clinical course over time.

## Supporting Information

File S1 **This document contains the clinical protocol for data collection, a description of the methods used to relate acoustic properties to peak flow rate, and the supplementary tables, Tables S1 & S2.**
(DOC)

Figure S1 **The figure shows an individual's inhaler daily use, peak flow recordings and AQLQ over a 90 day period.** The patient shows a progressive improvement in PEFR and AQLQ over the time, during which they demonstrate excellent adherence. The figure also shows that having achieved optimal PEFR and AQLQ they show a more variable adherence rate.
(TIF)

Figure S2 **Experimental setup of equipment used to extract drug from the Diskus DPI is shown.**
(TIF)

Checklist S1 **CONSORT Checklist.**
(DOC)
