# Supplementary material for: A Method to Assess Adherence in Inhaler Use through Analysis of Acoustic Recordings of Inhaler Events
Source: PLoS One. 2014 Jun 6;9(6):e98701. doi: 10.1371/journal.pone.0098701 (PMC4048229; doi:10.1371/journal.pone.0098701)
Supplement: File S1 — This document contains the clinical protocol for data collection, a description of the methods used to relate acoustic properties to peak flow rate, and the supplementary tables, Tables S1 & S2. (DOC) [file pone.0098701.s001.doc]

**Supplementary Methods**

Study protocol

The subject’s clinical history, AQLQ, medication use and PEFR were recorded at baseline. Subjects were provided with an INCA enabled Diskus inhaler and a Peak Expiratory Flow Rate meter and a symptom diary. Their inhaler technique was observed and corrected by a trained research nurse, according to a 10 step checklist, shown in supplementary data Table 1, using the teach-to-goal method [16]. The subjects were instructed to use the inhaler with one inhaled dose, twice daily and to measure their PEFR in the morning and evening and record the results in the diary. At the end of the month, for three consecutive months, participants returned the inhaler and both PEFR diary and the recordings were downloaded from the INCA device for analysis. At each visit the subjects completed clinical assessments and inhaler use and technique were reviewed and errors corrected using the teach-back method.

**Objective measure of the acoustic profile of inhalation and exhalation**

An air vacuum was employed to simulate an inhalation. Varying flow rates, generated by the vacuum, were controlled by a rotameter (Figure S2) and the inhaler weighed to assess the mass of drug extracted during each simulated inhalation. The flow rates for this test were varied between 100L/Min to 40L/Min in steps of 10L/Min and from 40L/Min to 10L/Min in steps of 5L/Min. This procedure was repeated for varying durations, i.e. 0.5sec, 1s, 2s and 3 seconds to assess the affect of inhalation duration on mass extracted.

Exhalations into the inhaler may disperse the drug from the inhaler before inhalation. Exhalations were performed, at different forces, in the direction of the inhaler, at approximately 10cm from the mouthpiece of a Diskus inhaler with an INCA device attached. To determine the weight of drug dispersed, the inhaler was weighed before and after each exhalation. The acoustic energy generated during exhalation was related to the weight of drug dispersed.

| **Errors** | Month 1 | Month 2 | Month 3 | *p value comparing month 1 to 3* |
| --- | --- | --- | --- | --- |
| **All Patients** |  |  |  |  |
| **Temporal** | 17% | 16% | 19% | 0.52 |
| **Technique** | 8% | 6% | 5% | 0.26 |
| **Combined** | 26% | 21% | 22% | 0.4 |
| **AQLQ Improvers** |  |  |  |  |
| **Temporal** | 15% | 14% | 16% | 0.59 |
| **Technique** | 13% | 6% | 6% | 0.18 |
| **Combined** | 23% | 20% | 20% | 0.7 |
| **AQLQ Non-Improvers** |  |  |  |  |
| **Temporal** | 20% | 20% | 23% | 0.57 |
| **Technique** | 4% | 5% | 4% | 0.99 |
| **Combined** | 32% | 23% | 26% | 0.5 |
| **PEFR Improvers** |  |  |  |  |
| **Temporal** | 15% | 16% | 17% | 0.37 |
| **Technique** | 13% | 9% | 8% | 0.55 |
| **Combined** | 23% | 19% | 17% | 0.33 |
| **PEFR Non-Improvers** |  |  |  |  |
| **Temporal** | 15% | 16% | 18% | 0.37 |
| **Technique** | 9% | 7% | 6% | 0.46 |
| **Combined** | 22% | 22% | 25% | 0.5 |
|  |  |  |  |  |

**Table S1:** Proportion of errors from all INCA recordings for all patients, patients with improvement in AQLQ and patients with improvement in PEFR; AQLQ = Asthma Quality of Life Questionnaire, PEFR = Peak Expiratory Flow Rate.

| **Adherence Rates** | Month 1 | Month 2 | Month 3 | *p value comparing month 1 to 3* |
| --- | --- | --- | --- | --- |
| **All Patients** |  |  |  |  |
| **Temporal** | 0.87 | 0.9 | 0.88 | *0.94* |
| **Technique** | 0.9 | 0.95 | 0.97 | ***0.03* |
| **Combined** | 0.78 | 0.86 | 0.84 | *0.24* |
| **AQLQ Improvers** |  |  |  |  |
| **Temporal** | 0.88 | 0.88 | 0.86 | *0.52* |
| **Technique** | 0.85 | 0.92 | 0.94 | ***0.056* |
| **Combined** | 0.74 | 0.84 | 0.83 | *0.15* |
| **AQLQ Non-Improvers** |  |  |  |  |
| **Temporal** | 0.9 | 0.93 | 0.88 | *0.67* |
| **Technique** | 0.94 | 0.94 | 0.96 | *0.53* |
| **Combined** | 0.86 | 0.88 | 0.85 | *0.95* |
| **PEFR Improvers** |  |  |  |  |
| **Temporal** | 0.85 | 0.87 | 0.86 | *0.85* |
| **Technique** | 0.88 | 0.92 | 0.91 | *0.54* |
| **Combined** | 0.75 | 0.84 | 0.85 | *0.16* |
| **PEFR Non-Improvers** |  |  |  |  |
| **Temporal** | 0.94 | 0.93 | 0.91 | *0.55* |
| **Technique** | 0.91 | 0.93 | 0.96 | *0.21* |
| **Combined** | 0.86 | 0.91 | 0.87 | *0.83* |
|  |  |  |  |  |

**Table S2:** Adherence Rates over study period for all patients, patients with improvement in AQLQ and patients with improvement in PEFR; AQLQ = Asthma Quality of Life Questionnaire, PEFR = Peak Expiratory Flow Rate.
