# Supplementary figures and images for: A Method to Assess Adherence in Inhaler Use through Analysis of Acoustic Recordings of Inhaler Events
Source: PLoS One. 2014 Jun 6;9(6):e98701. doi: 10.1371/journal.pone.0098701 (PMC4048229; doi:10.1371/journal.pone.0098701)

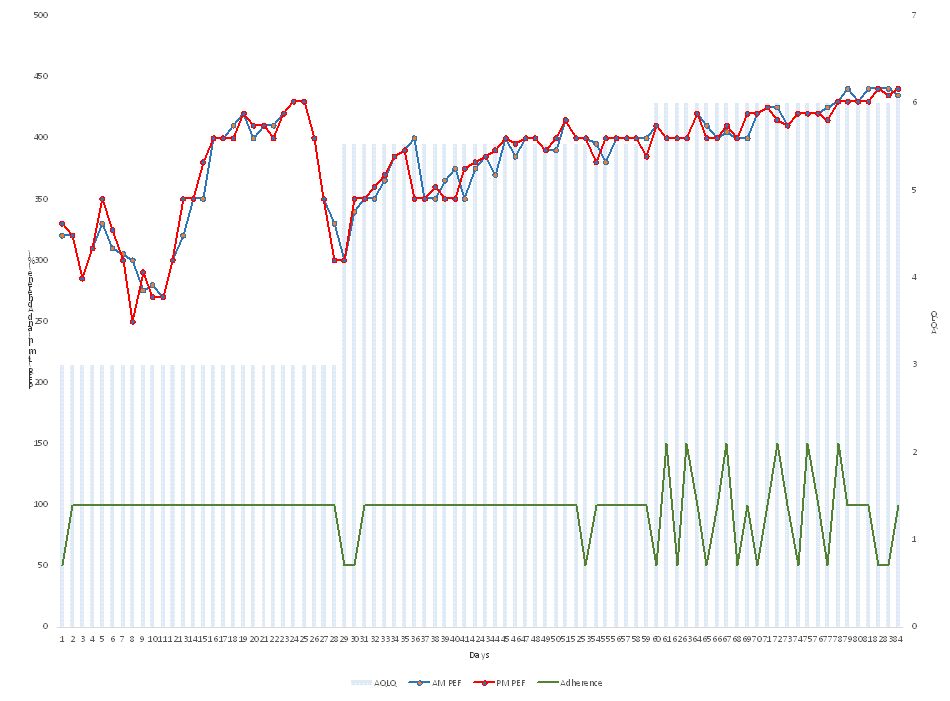

Supplement: Figure S1 — The figure shows an individual's inhaler daily use, peak flow recordings and AQLQ over a 90 day period. The patient shows a progressive improvement in PEFR and AQLQ over the time, during which they demonstrate excellent adherence. The figure also shows that having achieved optimal PEFR and AQLQ they show a more variable adherence rate. (TIF) [file pone.0098701.s002.tif]

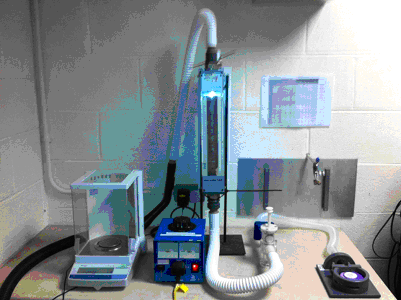

Supplement: Figure S2 — Experimental setup of equipment used to extract drug from the Diskus DPI is shown. (TIF) [file pone.0098701.s003.tif]
